# Safety and outcomes of different endovascular treatment techniques for anterior circulation ischaemic stroke in the elderly: data from the Imperial College Thrombectomy Registry

**DOI:** 10.1007/s00415-023-12077-3

**Published:** 2023-11-20

**Authors:** Lucio D’Anna, Lorenzo Barba, Matteo Foschi, Michele Romoli, Samir Abu-Rumeileh, Tsering Dolkar, Orsolya Vittay, Luke Dixon, Paul Bentley, Zoe Brown, Charles Hall, Omid Halse, Sohaa Jamil, Harri Jenkins, Dheeraj Kalladka, Joseph Kwan, Abid Malik, Maneesh Patel, Neil Rane, Dylan Roi, Abhinav Singh, Marius Venter, Soma Banerjee, Kyriakos Lobotesis

**Affiliations:** 1 Stroke Centre, Department of Stroke and Neuroscience, Charing Cross Hospital, Imperial College London NHS Healthcare Trust, Fulham Palace Road, London, W6 8RF UK; 2 Department of Brain Sciences, Imperial College London, London, UK; 3 Department of Neurology, Martin-Luther-University Halle-Wittenberg, Halle (Saale), Germany; 4 Department of Biotechnological and Applied Clinical Sciences, University of L’Aquila, L’Aquila, Italy; 5 Neurology and Stroke Unit, Department of Neuroscience, Bufalini Hospital, AUSL Romagna, Cesena, Italy; 6 Neuroradiology, Department of Imaging, Charing Cross Hospital, Imperial College London, NHS Healthcare Trust, London, UK

**Keywords:** Mechanical thrombectomy, Ischemic stroke, Technique, Elderly

## Abstract

**Background:**

Although previous studies investigated the main predictors of outcomes after endovascular thrombectomy (EVT) in patients aged 80 years and older, less is known about the impact of the procedural features on outcomes in elderly patients. The aim of this study was to investigate the influence of EVT technical procedures on the main 3-month outcomes in a population of patients aged 80 years and older.

**Methods:**

This observational, prospective, single-centre study included consecutive patients with acute LVO ischaemic stroke of the anterior circulation. The study outcomes were functional independence at 3 months after EVT (defined as a mRS score of 0–2), successful reperfusion (mTICI ≥ 2b), incidence of haeamorrhagic transformation, and 90-day all cause of mortality.

**Results:**

Our cohort included 497 patients with acute ischaemic stroke due to LVO treated with EVT. Among them, 105 (21.1%) patients were aged ≥ 80 years. In the elderly group, multivariable regression analysis showed that thromboaspiration technique vs stent-retriever was the single independent predictor of favourable post-procedural TICI score (OR = 7.65, 95%CI = 2.22–26.32, *p* = 0.001).

**Conclusions:**

Our study suggests that EVT for LVO stroke in the elderly could be safe. The use of thromboaspiration was associated with positive reperfusion outcome in this population. Further studies in larger series are warranted to confirm the present results and to evaluate the safety and efficacy of EVT in the elderly and oldest adults.

## Introduction

Endovascular thrombectomy (EVT) currently represents the standard of care of acute ischaemic stroke due to large vessel occlusion (LVO) irrespective of the age of the patient [1]. Elderly patients (aged ≥ 80 or older) account for a major proportion of ischaemic strokes worldwide and the use of EVT in the elderly has been increasing in recent years. Major clinical trials have demonstrated the benefit of EVT for the general population although the degree of benefit is less clear in the elderly populations [2–4]. Two recent large meta-analyses reported higher rates of worse functional outcomes at 90 days and lower rate of successful recanalization after EVT in elderly patients compared to the non-elderly group [5, 6]. Although EVT is not associated with an increased rates of post-procedural symptomatic intracerebral haemorrhage (sICH) compared to younger subjects [5], the efficacy and the cost-effectiveness of EVT in the elderly group is still debated [7].

Previous studies investigated the main predictors of outcomes in patients aged 80 years and older after EVT [8, 9]. Beuker et al. evaluated in a large ‘real-world’ cohort the clinically relevant comorbidities associated with outcomes in elderly patients with stroke treated with EVT [8]. The authors found that diabetes mellitus, previous myocardial infarction, chronic kidney disease and dementia were significantly associated with risk of disability and death after EVT. Tiainen et al. showed that in logistic regression analysis higher admission NIHSS score, not performing thrombolysis, lack of recanalization and higher frailty status were all independently associated with very poor outcomes at 3 months [9]. They also found that male gender, not performing thrombolysis, sICH, and higher frailty status were factors associated with 1-year mortality. To date only few studies have investigated the impact of the procedural features on outcomes after EVT in elderly patients [10–12]. Also, the device choice for the first thrombectomy pass and the success rates of the EVT techniques in elderly patients are still unclear [13]. The aim of this study was to investigate the influence of EVT technical procedures on the main 3-month outcomes in a population of patients aged 80 years and older.

## Methods

In this observational, investigator-initiated, prospective study, all acute stroke patients consecutively treated with EVT at the Stroke Department, Charing Cross Hospital, Imperial College Healthcare NHS Trust, London between 1st January 2016 and 30th June 2021 were included. This study has obtained approval from the UK Health Regulator Authority (HRA) (HRA Reference No.: 275260). The study has also received confirmation of capacity and capability from the Imperial College Healthcare NHS Trust. The study was conducted in accordance with the recommendations for physicians involved in research on human subjects adopted by the 18th World Medical Assembly, Helsinki 1964 and later revisions. The Stroke Department at Charing Cross Hospital is the Northwest London (UK) regional Comprehensive Stroke Center (CSC) for MT in an urban metropolitan area with more than 6.4 million people. Please refer to our previous manuscripts for the organization of the Imperial Stroke Thrombectomy network [14, 15]. In 2019, the life expectancy at birth in London was 85.4 years for women and 81.6 years for men [16].

### Patient inclusion and exclusion criteria for the analysis

For the purpose of this analysis, the criteria for patient selection were: (1) age ≥ 18 years; (2) NIHSS score of 6 or more; (3) Alberta Stroke Program Early CT score (ASPECTS) of 5 or more; (4) LVO sites: distal internal intracranial carotid artery, middle cerebral artery segments M1 or M2; (5) initiation of the EVT had to be possible within 6 h after the stroke onset (6) modified Rankin scale (mRS) pre event 0–2. Intravenous thrombolysis (IVT) was administered in all patients who presented within 4.5 h of stroke symptom onset without contraindications according to guidelines. For this analysis, we excluded stroke patients with basilar artery occlusion and patients that met DAWN or DEFUSE 3 eligibility criteria [3, 4]. Stroke aetiology was evaluated according to the use of Trial of ORG 10172 in Acute Stroke Treatment (TOAST) criteria by a study neurologist (L.D) [17, 18]^.^

### Endovascular treatment

The Imperial Stroke Centre registry prospectively collected data of consecutive patients treated with EVT and encompassed patient characteristics, including age, vascular risk factors, laboratory results, and relevant medical history. The prescription of treatments before admission was recorded. The choice of treatment was decided by the treating physician as part of routine clinical care pre-admission. NIHSS was performed in all patients on admission and 24 h after the MT. Early neurological improvement was defined as improvement by ≥ 4 points at 24 h. The modified Rankin Scale (mRS) was used to assess the patient’s initial pre-stroke status and the level of functional independence at 90 days of the patients was evaluated centrally through a telemedicine consultation or in-person consultation. Procedural metrics were collected prospectively. The extent of the initial core infarct was determined on pre-therapeutic CT using ASPECTS. In addition, an independent rater (consultant neuroradiologist) who did not participate in the endovascular stroke treatment of included patients, evaluated pre-therapeutic CT, and follow-up CT at 24 h to track a possible haemorrhagic transformation. Procedures were performed using third generation thrombectomy devices (aspiration catheter or stent retriever or both), and validated modalities were left to the discretion of the operator. Choice of local or general anaesthesia depended on medical condition and the need for general anaesthesia. At the end of the procedure, the interventional neuroradiologist attributed a modified Thrombolysis in Cerebral Infarction (mTICI) score. A successful reperfusion was defined as mTICI ≥ 2b grade after the endovascular procedure [19].

### Outcomes definitions

The prespecified outcomes of the present study were functional independence at 3 months after EVT defined as a mRS score of 0–2. Other outcomes were successful reperfusion (mTICI ≥ 2b), incidence of haemorrhagic transformation (sICH; according to Safe Implementation of Thrombolysis in Stroke-Monitoring Study [SITS-MOST], European Cooperative Acute Stroke Study-II [ECASS-II], Solitaire With the Intention for Thrombectomy as Primary Endovascular Treatment [SWIFT- PRIME]) and 90-day all cause of mortality.

### Statistical analysis

All statistical analyses were conducted with R 4.2.2 (R foundation, Vienna, Austria) with the following packages: *dplyr, lme4, stats, jtools*. To compare continuous variables between two or more groups, we adopted the Mann–Whitney *U* test and the Kruskal–Wallis test (followed by Dunn–Bonferroni post-hoc test), respectively. For categorical variables, we used the χ^2^ test. Associations between demographic, clinical or interventional variables and binary outcomes (see below) were tested with uni- and multivariable logistic regression models. We included in the multivariable analysis all variables that tested significant at univariable analysis (*p* < 0.05). Binary outcomes were defined as follows: favourable (mRS of 0–2) vs. unfavourable (mRS of 3–6) functional outcome at 3-month follow-up; non-survival vs. survival after 90 days; post-interventional haemorrhagic transformation yes vs. no; successful (mTICI score of 2b, 2c or 3) vs. unsuccessful revascularization (mTICI score of 0, 1 or 2a). Results of regression analysis are reported as odds ratio (OR) with 95% confidence interval (95%), *z*-values and *p*-values. Statistical significance was set at *p*-values < 0.05.

## Results

During the study period, 497 patients with acute ischaemic stroke and LVO of the anterior circulation underwent EVT in our centre. Among them, 392 (78.9%) and 105 (21.1%) patients were aged < 80 years and ≥ 80 years, respectively. The demographical and clinical data of the elderly and non-elderly groups are presented in Table 1. Overall, the mean age was 66.7 years (standard deviation, ± 14.4 years) and 45.1% were female (*n* = 224). The mean ages were 61.9 years (standard deviation, ± 12.2 years) and 84.5 years (standard deviation, ± 4.0 years) in the non-elderly and elderly group, respectively. The two groups differed significantly in regards of the smoking status (*p* = 0.002). Elderly patients more frequently were on treatment with statins (*p* = 0.018) and blood pressure-lowering drugs (*p* < 0.001) before the index event compared to non-elderly patients. Moreover, the percentage of patients with hypertension (*p* < 0.001) and atrial fibrillation (*p* < 0.001) was significantly higher in the elderly group than in the non-elderly group. Elderly patients suffered more often than non-elderly patients of cardioembolic strokes (*p* < 0.001).

**Table 1:** Demographic and clinical characteristics

	Overall cohort (*n* = 497)	Non-elderly < 80 years (*n* = 392)	Elderly ≥ 80 years (*n* = 105)	*p*
Age, years [mean ± SD]	66.7 ± 14,4	61.9 ± 12,2	84.5 ± 4.0	** < 0.001**
Female sex [*n*, (%)]	224 (45.1)	169 (43.1)	55 (52.3)	0.113
Smoking status [*n*, (%)]				
Never smoker	389 (78.3)	295 (75.3)	94 (89.5)	**0.002**
Current smoking	77 (15.5)	72 (18.3)	5 (4.8)	
Former smoking	31 (6.2)	25 (6.4)	6 (5.7)	
NIHSS at admission [median (IQR)]	17 (13–21)	17 (13–21)	18 (14–22)	0.051
ASPECTS at admission [median (IQR)]	8 (7–9)	8 (7–9)	8 (7–9)	0.851
Therapy on admission				
Anticoagulation [*n*, (%)]	84 (16.9)	61 (15.6)	23 (21.9)	0.163
Antiplatelet drugs [*n*, (%)]	116 (23.3)	93 (23.7)	23 (21.9)	0.951
Statins [*n*, (%)]	167 (33.6)	121 (30.9)	46 (43.8)	**0.018**
Blood pressure-lowering drugs [*n*, (%)]	245 (49.3)	176 (44.9)	69 (65.7)	**< 0.001**
Previous diseases				
Hypertension [*n*, (%)]	268 (53.9)	193 (49.2)	75 (71.4)	**< 0.001**
Diabetes mellitus [*n*, (%)]	96 (19.3)	75 (19.1)	21 (20.0)	0.915
Hypercholesterolemia [*n*, (%)]	218 (43.9)	166 (42.3)	52 (49.5)	0.228
Coronary artery disease [*n*, (%)]	82 (16.5)	62 (15.8)	20 (19.1)	0.519
Congestive heart failure [*n*, (%)]	46 (9.3)	32 (8.2)	14 (13.3)	0.151
Symptomatic carotid artery disease [*n*, (%)]	51 (10.2)	42 (10.7)	9 (8.6)	0.644
Previous TIA/ischemic stroke [*n*, (%)]	91 (18.3)	70 (17.9)	21 (20.0)	0.717
Atrial fibrillation [*n*, (%)]	206 (41.4)	140 (35.7)	66 (62.9)	**< 0.001**
Previous history	106 (21.3)	69 (17.6)	37 (35.3)	**< 0.001**
Newly diagnosed	100 (20.1)	71 (18.1)	29 (27.6)	**0.043**
Stroke etiology				
Cardioembolic	209 (42.0)	145 (37.0)	64 (61.0)	**< 0.001**
LAA	47 (9.5)	38 (9.7)	9 (8.5)	
Other causes	6 (1.2)	5 (1.3)	1 (1.0)	
Undetermined aetiology	235 (47.3)	204 (52.0)	31 (29.5)	
Tandem occlusion (ICA + M1) [*n*, (%)]				
Yes	100 (20.1)	85 (21.7)	15 (14.3)	0.123
No	397 (79.9)	307 (78.3)	90 (85.7)	

*ASPECTS* The Alberta Stroke Program Early CT Score, *DAPT* dual antiplatelet therapy, *DOAC* direct oral anticoagulant, *ICA* internal carotid artery, *mRS* modified Rankin Scale, *LAA* large artery atherosclerosis, *LMWH* low molecular weight heparin, *NIHSS* National Institutes of Health Stroke Scale, *TICI* thrombolysis in cerebral infarction scale

Significance at *p* < 0.05

Table 2 shows the comparison of the procedural features between the elderly and non-elderly groups. The two groups did not differ in terms of stroke prehospital pathway, hyperacute management, type of anaesthesia used, time intervals, number of passes, and first pass effect. The elderly group compared to the non-elderly group did not differ also in terms of type of endovascular technique used. The percentage of patients with early neurological improvement was significantly higher in the non-elderly group (57.3% vs 45.6%, *p* = 0.046). Supplemental Table 1 reports usage of different endovascular techniques and the type of anaesthesia during the study period. From 1st January 2016 to 30th June 2021, we observed a significant difference in the use of different endovascular techniques and the type of anaesthesia.

**Table 2:** Procedural features

	Overall cohort (*n* = 497)	< 80 years (*n* = 392)	≥ 80 years (*n* = 105)	*p*
Stroke prehospital pathway [*n*, (%)]				
Mothership	323 (65.0)	255 (65.0)	68 (65.0)	1.00
Drip and ship	174 (35.0)	137 (35.0)	37 (35.0)	
Hyperacute treatment [*n*, (%)]				
EVT alone	129 (26.0)	101 (25.8)	28 (26.7)	0.950
EVT + IVT	368 (74.0)	291 (74.2)	77 (73.3)	
Type of anaesthesia [*n*, (%)]				
LA	187 (37.6)	148 (37.7)	39 (37.1)	0.375
GA	303 (61.0)	237 (60.5)	66 (62.9)	
Converted from LA to GA	7 (1.4)	7 (1.8)	0 (0.0)	
Time intervals in minutes				
Onset to needle [median (IQR)]	124 (92–160)	122 (92–160)	127 (96–166)	0.450
Onset to groin puncture [median (IQR)]	280 (227–330)	280 (226–328)	286 (235–331)	0.492
Onset to reperfusion [median (IQR)]	330 (290–347)	324 (303–348)	334 (273–338)	0.201
Endovascular therapy [*n*, (%)]				
SR alone	93 (18.7)	74 (18.9)	19 (18.1)	0.951
TAS alone	263 (52.9)	206 (52.5)	57 (54.3)	
TAS + SR	141 (28.4)	112 (28.6)	29 (27.6)	
N. of passes [*n*, (%)]*				
1	187 (38.9)	154 (40.7)	33 (32.0)	0.238
2	134 (27.9)	104 (27.5)	30 (29.1)	
3 or more	160 (33.2)	120 (31.8)	40 (38.9)	
First pass effect [*n*, (%)]*	180 (36.2)	149 (38)	31 (29.5)	0.210

*CSC* comprehensive stroke center, *GA* general anaesthesia, *IVT* intravenous thrombolysis, *LA* local anaesthesia, *EVT* endovascular treatment, *SR* stent retriever, *TAS* thromboaspiration. *Data not available for 16 patients

A comparison of the study outcomes between the elderly and non-elderly groups is shown in Table 3. The proportion of patients with functional independence at 90 days after the stroke was significantly higher in the non-elderly group (45.2% vs 19%, *p* < 0.001). Patients in the elderly group had a significantly lower rate of post-intervention favourable TICI score (76.2% vs 86.2%, *p* = 0.019). No differences between the two groups were found in terms of mortality at 90 days and rate of haemorrhagic transformations post EVT.

**Table 3:** Study outcomes

	Overall cohort (*n* = 497)	< 80 years (*n* = 392)	≥ 80 years (*n* = 105)	*p*-value
90-day mRS [*n*, (%)]				
≤ 2	197 (59.6)	177 (45.2)	20 (19.0)	**< 0.001**
> 2	300 (60.4)	215 (54.8)	85 (81.0)	
∆ NIHSS 24 h [*n*, (%)]*				
< 4	221 (45.2)	165 (42.7)	56 (54.4)	**0.046**
≥ 4	268 (54.8)	221 (57.3)	47 (45.6)	
Post-intervention TICI [*n*, (%)]				
Favourable (2b, 2c, 3)	418 (84.1)	338 (86.2)	80 (76.2)	**0.019**
Unfavourable (0, 1, 2a)	79 (15.9)	54 (13.8)	25 (23.8)	
Death at 90 days [*n*, (%)]	49 (9.9)	37 (9.4)	12 (11.4)	0.672
Early post-procedural complications				
HT [*n*, (%)]	114 (22.9)	92 (23.5)	22 (21.0)	0.679
1a	25 (5.0)	18 (4.6)	7 (6.7)	
1b	30 (6.0)	30 (7.7)	0 (0.0)	
1c	7 (1.4)	6 (1.5)	1 (1.0)	
2	30 (6.0)	20 (5.1)	10 (9.5)	
3a	3 (0.6)	3 (0.8)	0 (0.0)	
3b	4 (0.8)	4 (1.0)	0 (0.0)	
3c	15 (3.0)	11 (2.8)	4 (3.8)	
PH (HT 1c, 2, 3a, 3b) [*n*, (%)]	44 (8.9)	33 (8.4)	11 (10.5)	0.284
SAH (HT 3c) [*n*, (%)]	15 (3.0)	11 (2.8)	4 (3.8)	0.832
sICH [*n*, (%)]	25 (5.0)	22 (5.6)	3 (2.9)	0.370
sICH and/or PH [*n*, (%)]	56 (11.3)	43 (11.0)	13 (12.4)	0.507

*HT* haemorrhagic transformation, *MCA* middle cerebral artery, *mRS* modified Rankin Scale, *PH* parenchymal hematoma, *SAH* subarachnoid haemorrhage, *sICH* symptomatic intracranial haemorrhage, *TICI* thrombolysis in cerebral infarction scale

*8 patients (6 < 80 years and 2 > 80 years) died before 24 h, thus no 24 h NIHSS was collected

Significance at *p* < 0.05

Table 4 shows the logistic regression analysis to determine the predictors of functional independence at 3 months after EVT in the elderly patients. Multivariable regression analysis showed that only early neurological improvement was the only independent predictor of good functional outcome at 90 days in elderly patients.

**Table 4:** Logistic regression analysis for mRS 0–2 at 90-day in patients ≥ 80 years of age

	≥ 80 years (*n* = 105)					
	Univariate analysis			Multivariate analysis		
	OR (95% CI)	*z*	*p*	OR (95% CI)	*z*	*p*
Age (per 1 year increase)	1.05 (0.94–1.19)	0.87	0.383	–	–	–
Female sex	0.89 (0.34–2.36)	− 0.24	0.812	–	–	–
Current vs. never smoker*	–	–	–	–	–	–
NIHSS on admission (per 1 point increase)	0.92 (0.83–1.01)	− 1.76	0.079	–	–	–
ASPECTS at admission CT (per 1 point increase)	1.14 (0.77–1.69)	0.64	0.521	–	–	–
Tandem lesion (ICA + M1) (yes vs. no)	0.62 (0.13–2.97)	− 0.60	0.546	–	–	–
Stroke aetiology (LAA vs. cardioembolic)	0.45 (0.05–3.88)	− 0.73	0.464	–	–	–
Therapy on admission						
Anticoagulation	1.24 (0.40–3.87)	0.37	0.710	–	–	–
Antiplatelet drugs	0.57 (0.15–2.16)	− 0.82	0.411	–	–	–
Statins	0.82 (0.31–2.22)	− 0.38	0.703	–	–	–
Blood pressure-lowering drugs	1.27 (0.44–3.65)	0.45	0.654	–	–	–
Previous diseases						
Hypertension	0.69 (0.24–1.94)	− 0.70	0.481	–	–	–
Diabetes	1.44 (0.46–4.54)	0.62	0.536	–	–	–
Hypercholesterolaemia	1.69 (0.63–4.55)	1.03	0.301	–	–	–
Coronary artery disease	0.71 (0.19–2.69)	− 0.51	0.610	–	–	–
Congestive heart failure	0.68 (0.14–3.29)	− 0.48	0.628	–	–	–
Symptomatic carotid artery disease	0.51 (0.06–4.30)	− 0.62	0.533	–	–	–
Previous ischemic stroke	0.39 (0.05–3.28)	− 0.86	0.389	–	–	–
Previous TIA/ischaemic stroke	0.66 (0.17–2.49)	− 0.62	0.537	–	–	–
Atrial fibrillation (known or newly diagnoses)	1.48 (0.52–4.24)	0.73	0.464	–	–	–
Mothership pre-hospital system of care	0.99 (0.36–2.74)	− 0.02	0.980	–	–	–
Hyperacute treatment (EVT + IVT vs. EVT alone)	1.57 (0.48–5.19)	0.74	0.456	–	–	–
Type of anaesthesia (GA vs. LA)	0.40 (0.15–1.08)	− 1.80	0.071	–	–	–
Onset-to-needle time (per 1 min increase)	1.003 (0.995–1.01)	0.92	0.356	–	–	–
Onset-to-groin time (per 1 min increase)	0.999 (0.99–1.006)	− 0.35	0.727	–	–	–
EVT technique						
TAS alone vs. SR alone**	–	–	–	–	–	–
N. of passes	0.66 (0.36–1.23)	− 1.31	0.192	–	–	–
Post-interventional TICI (0,1, 2a vs. 2b, 2c, 3)	0.30 (0.06–1.39)	− 1.54	0.124	–	–	–
24 h NIHSS change < 4	0.15 (0.05–0.49)	− 3.16	**0.002**	0.26 (0.08–0.91)	− 2.11	**0.035**
Post-interventional HT	0.36 (0.08–1.69)	− 1.29	0.196	–	–	–

*ASPECTS* Alberta Stroke Program Early CT Score, *GA* general anaesthesia, *HT* haemorrhagic transformation, *IVT* intravenous thrombolysis, *LA* local anaesthesia, *mRS* modified Rankin Scale, *EVT* endovascular treatment, *NIHSS* National Institutes of Health Stroke Scale, *OR* odds ratio, *TIA* transient ischemic attack, *TICI* thrombolysis in cerebral infarction scale, *SR* stent retriever, *TAS* thromboaspiration

*All current smokers aged ≥ 80 had 90-day mRS 3–6

**All patients aged ≥ 80 who were treated with TAS alone had 90-day mRS 0–2

Significance at *p* < 0.05

Table 5 shows the logistic regression analysis to determine the predictors of successful post-procedural TICI score. In the elderly group, the single-variable regression and multivariable regression analysis showed that thromboaspiration technique vs stent-retriever was the single independent predictor of favourable post-procedural TICI score (OR = 7.65, 95%CI = 2.22–26.32, *p* = 0.001).

**Table 5:** Logistic regression analysis for favourable post-procedural TICI score (2b, 2c, 3) in patients ≥ 80 years of age

	≥ 80 years (*n* = 105)					
	Univariate analysis			Multivariate analysis		
	OR (95% CI)	*z*	*p*	OR (95% CI)	*z*	*p*
Age (per 1 year increase)	1.08 (0.96–1.23)	1.25	0.211	–	–	–
Female sex	0.67 (0.27–1.66)	− 0.87	0.384	–	–	–
Current vs. never smoker	0.43 (0.07–2.75)	− 0.89	0.374	–	–	–
NIHSS on admission (per 1 point increase)	1.0006 (0.92–1.09)	0.01	0.990	–	–	–
ASPECTS at admission CT (per 1 point increase)	0.94 (0.67–1.31)	− 0.39	0.699	–	–	–
Tandem lesion (ICA + M1) (yes vs. no)	2.23 (0.47–10.6)	1.00	0.314	–	–	–
Stroke aetiology (LAA vs. cardioembolic)	2.24 (0.26–19.46)	0.73	0.464	–	–	–
Therapy at admission						
Anticoagulation	0.85 (0.30–2.75)	− 0.29	0.772	–	–	–
Antiplatelet drugs	1.16 (0.38–3.53)	0.26	0.792	–	–	–
Statins	1.53 (0.61–3.87)	0.90	0.369	–	–	–
Blood pressure-lowering drugs	2.15 (0.86–5.40)	1.64	0.102	–	–	–
Previous diseases						
Hypertension	0.96 (0.36–2.62)	-0.07	0.942	–	–	–
Diabetes	1.00 (0.33–3.07)	0.00	1.00	–	–	–
Hypercholesterolaemia	1.08 (0.44–2.66)	0.17	0.861	–	–	–
Coronary artery disease	1.31 (0.39–4.36)	0.44	0.657	–	–	–
Congestive heart failure	0.51 (0.15–1.68)	− 1.11	0.268	–	–	–
Symptomatic carotid artery disease	2.67 (0.32–22.43)	0.90	0.367	–	–	–
Previous ischemic stroke	0.81 (0.20–3.34)	− 0.28	0.776	–	–	–
Previous TIA/ischaemic stroke	1.42 (0.43–4.68)	0.57	0.568	–	–	–
Atrial fibrillation (known or newly diagnoses)	1.17 (0.47–2.94)	0.34	0.735	–	–	–
Procedural features						
Mothership pre-hospital system of care	0.76 (0.30–1.93)	− 0.57	0.569	–	–	–
Hyperacute treatment (EVT + IVT vs. EVT alone)	0.83 (0.29–2.36)	− 0.35	0.730	–	–	–
Type of anaesthesia (GA vs. LA)	1.17 (0.47–2.94)	0.34	0.735	–	–	–
Onset-to-needle time (per 1 min increase)	0.99 (0.98–1.0002)	− 1.90	0.057	–	–	–
Onset-to-groin time (per 1 min increase)	0.99 (0.988–1.001)	− 1.66	0.097	–	–	–
TAS alone vs. SR alone	7.65 (2.22–26.32)	3.23	**0.001**	2.34 (0.58–9.38)	1.20	0.232
N. of passes	0.38 (0.21–0.69)	− 3.16	**0.002**	0.20 (0.07–0.59)	− 2.92	**0.004**

*ASPECTS* Alberta Stroke Program Early CT Score, *GA* general anaesthesia, *HT* haemorrhagic transformation, *IVT* intravenous thrombolysis, *LA* local anaesthesia, *mRS* modified Rankin Scale, *EVT* endovascular treatment, *NIHSS* National Institutes of Health Stroke Scale, *OR* odds ratio, *TIA* transient ischemic attack, *TICI* thrombolysis in cerebral infarction scale, *SR* stent retriever, *TAS* thromboaspiration

Significance at *p* < 0.05

Table 6 describes the logistic regression analysis to determine the predictors of haemorrhagic transformation post EVT. In the elderly group hypertension, diabetes and history of previous ischaemic stroke were statistically significant indicators of haemorrhagic transformation post-EVT. Multivariable regression analysis incorporating all these indicators showed that diabetes was the only independent predictor of haemorrhagic transformation post EVT in the elderly.

**Table 6:** Logistic regression analysis for post-procedural haemorrhagic transformation in patients ≥ 80 years of age

	≥ 80 years (*n* = 105)					
	Univariate analysis			Multivariate analysis		
	OR (95% CI)	*z*	*p*	OR (95% CI)	*z*	*p*
Age (per 1 year increase)	1.03 (0.92–1.15)	0.48	0.635	–	–	–
Female sex	0.70 (0.27–1.81)	− 0.73	0.466	–	–	–
Current vs. never smoker	0.67 (0.25–1.75)	− 0.06	0.946	–	–	–
NIHSS on admission (per 1 point increase)	1.08 (0.97–1.19)	1.45	0.147	–	–	–
ASPECTS at admission CT (per 1 point increase)	0.85 (0.60–1.22)	− 0.86	0.388	–	–	–
Tandem lesion (ICA + M1) (yes vs. no)	0.93 (0.24–3.65)	− 0.10	0.922	–	–	–
Stroke aetiology (LAA vs. cardioembolic)	2.17 (0.47–9.92)	1.00	0.319	–	–	–
Therapy at admission						
Anticoagulation	1.95 (0.68–5.58)	1.25	0.211	–	–	–
Antiplatelet drugs	2.59 (0.92–7.28)	1.81	0.071	–	–	–
Statins	1.37 (0.53–3.52)	0.66	0.511	–	–	–
Blood pressure-lowering drugs	1.51 (0.53–4.27)	0.78	0.438	–	–	–
Previous diseases						
Hypertension	5.09 (1.11–23.35)	2.09	**0.036**	3.41 (0.71–16.42)	1.53	0.126
Diabetes	5.45 (1.90–15.62)	3.16	**0.002**	4.09 (1.36–12.25)	2.51	**0.012**
Hypercholesterolaemia	1.29 (0.50–3.31)	0.53	0.597	–	–	–
Coronary artery disease	1.85 (0.62–5.55)	1.09	0.274	–	–	–
Congestive heart failure	1.03 (0.26–4.08)	0.04	0.963	–	–	–
Symptomatic carotid artery disease	2.03 (0.46–8.85)	0.94	0.348	–	–	–
Previous ischemic stroke	2.41 (0.64–9.14)	1.30	0.195	–	–	–
Previous TIA/ischemic stroke	3.08 (1.08–8.80)	2.10	**0.036**	2.23 (0.71–6.98)	1.38	0.167
Atrial fibrillation (known or newly diagnoses)	0.82 (0.31–2.14)	− 0.41	0.681	–	–	–
Procedural features						
Mothership pre-hospital system of care	2.19 (0.84–5.70)	1.61	0.108	–	–	–
Hyperacute treatment (EVT + IVT vs. EVT alone)	0.56 (0.20–1.52)	− 1.15	0.251	–	–	–
Type of anaesthesia (GA vs. LA)	1.34 (0.49–3.66)	0.58	0.562	–	–	–
Onset-to-needle time (per 1 min increase)	0.99 (0.98–1.005)	− 1.22	0.223	–	–	–
Onset-to-groin time (per 1 min increase)	0.99 (0.988–1.001)	− 1.47	0.141	–	–	–
TAS alone vs. SR alone	0.70 (0.19–2.61)	− 0.53	0.599	–	–	–
N. of passes	1.36 (0.81–2.29)	1.17	0.244	–	–	–
Post-procedural variables						
Post-interventional TICI (0,1, 2a vs. 2b, 2c, 3)	1.26 (0.43–3.68)	0.43	0.668	–	–	–
24 h NIHSS change < 4	2.73 (0.97–7.69)	1.91	0.057	–	–	–

*ASPECTS* Alberta Stroke Program Early CT Score, *GA* general anaesthesia, *HT* haemorrhagic transformation, *IVT* intravenous thrombolysis, *LA* local anaesthesia, *mRS* modified Rankin Scale, *EVT* endovascular treatment, *NIHSS* National Institutes of Health Stroke Scale, *OR* odds ratio, *TIA* transient ischemic attack, *TICI* thrombolysis in cerebral infarction scale, *SR* stent retriever, *TAS* thromboaspiration

Significance at *p* < 0.05

Finally, supplemental Table 2 shows the logistic regression analysis to determine the predictors of 3-month mortality after EVT. In the elderly-group single-variable regression and multivariable regression analysis showed that only early neurological improvement post-EVT was an independent predictor of outcome of death at 90 days post-stroke.

## Discussion

Our analysis showed that the use of thromboaspiration technique alone is a major determinant of successful reperfusion post EVT in elderly patients. EVT has now been consolidated as the mainstay therapy for large vessel occlusion, together with systemic fibrinolysis [1]. However, even though a stroke is an age-related illness connected with the cardiovascular risk profile, patients aged 80 years or older have been often underrepresented in the major bigger clinical trials. Indeed, the HERMES meta-analysis included a limited sample size of only 198 patients aged over 80 years [2]. Moreover, very few studies investigated the impact of the type of thrombectomy technique (aspiration vs stent retriever) on clinical and reperfusion outcomes post EVT in the elderly. Lai et al. investigated the predictors of only 30-day mortality after EVT in 48 patients aged 80 years or more. The authors found that the type of technique for thrombectomy was not associated with an increased likelihood of death at 30 days post stroke. Our results are in line with the study of Lai et al. as we showed that the type of thrombectomy technique is not associated with risk of death after EVT [11]. However, in our sample of 105 patients aged 80 years or older we investigated not only the predictors of 90-day mortality, but also those of functional independence at 3 months, successful reperfusion (mTICI ≥ 2b) and haemorrhagic transformation. Our results partially confirm also the analysis made by Alawieh et al [12]. In this study, the multivariable logistic regression analysis revealed that the use of thromboaspiration was not an independent predictor either of good functional outcome (mRS 0–2), mortality at 90 days or successful mTICI post-EVT. Contrary to Alawieh et al., in our study the thromboaspiration technique was an independent predictor of successful reperfusion post EVT at the multivariable logistic regression analysis. Further data regarding the thrombectomy technique associated with the clinical and reperfusion outcomes in this subgroup of patients undergoing EVT are needed to understand the factors that might prevent these patients from achieving good outcomes.

Interestingly, the positive effect of thromboaspiration alone was observed also in the non-elderly group of patients. Based on our findings, we could not clarify the mechanism of higher favourable mTICI in patients treated with thromboaspiration technique. Previous retrospective studies have reported increased successful revascularization rates when using contact aspiration as first-line endovascular treatment compared to stent-retriever [20–23]. Conversely, the ASTER and COMPASS multicentre randomised controlled trials with patients with acute ischaemic stroke and LVO randomised to undergo either contact aspiration or stent retriever showed that first-line aspiration was not superior to first-line stent in achieving successful revascularization at the end of the endovascular procedure and in terms of 90-day functional outcome [24, 25]. In our cohort of patients, almost 53% of the patients were treated with aspiration technique alone while stent retriever was used in almost 19% of the cases treated. It is noteworthy to mention that in our study the choice of the thrombectomy procedures were left to the discretion and experience of the operator and were not dictated by a specific protocol. Although aspiration has not been demonstrated to be superior or noninferior to the stent retriever technique, in our centre aspiration technique has gained growing acceptance as it is thought to facilitate revascularization quickly and potentially at a lower cost. In cases in which aspiration alone is not successful in removing the thrombus, the large-bore aspiration catheter provides the additional benefit of offering access for a stent retriever if needed. However, controversies about the relevance of the thrombectomy technique have arisen in literature [13]. As stent retriever techniques were used predominantly in randomised clinical trials, questions remain regarding the safety and efficacy of aspiration thrombectomy techniques as a first-line therapy and whether there is a mechanical thrombectomy technique associated with an upper age limit that prevents a subgroup of patients from achieving a good outcome.

Finally, in our analysis we did not observe any differences between elderly and non-elderly patients in terms of mortality at 90 days and rate of haemorrhagic transformations post EVT. This finding is in line with a metanalysis based on five endovascular trial datasets showing no difference in the risk of parenchymal hematoma and sICH between population of different ages [26]. A more recent systematic review by Zhao et al. investigated differences in outcomes for patients 80 years or older compared to younger patients [6]. Data from 3954 patients across 16 studies from 2014 to 2019 showed that the rates of sICH in patients aged ≥ 80 years was found higher than those in patients aged < 80 but did not reach statistical significance. However, older patients showed to have higher odds of mortality at 90 days. Our data suggested that EVT is generally safe in a selected cohort of elderly patients. However, special considerations in terms of patient selection and management should be made by clinicians to select elderly patients for EVT. This requires a multimodal approach taking into account the baseline functional status of the patient, the infarct size, white matter disease burden, neuroimaging characteristics (e.g. evidence of poor collaterals) and technical considerations (e.g. vessel tortuosity) [13]. We believe that future clinical trials are needed to develop a systemic method incorporating various clinical and technical variables of EVT to optimise patient selection for EVT in the elderly population.

Our analysis had the following strengths: (1) data ascertainment was undertaken systematically and prospectively; (2) large cohorts of patients as a single centre study. Nevertheless, our study has several limitations. First, the non-randomised design of the study that might have introduced bias. Even though we adjusted for this factor in the logistic regression analyses to determine their impact on the outcomes this could represent a potential bias. Finally, this study was conducted as single centre study.

In conclusions, our study seems to suggest that EVT for LVO stroke in the elderly could be safe. Major determinants of reperfusion outcome in this population is the use of thromboaspiration technique. Further studies in larger series are warranted to confirm the results and to evaluate the safety and efficacy of EVT in the elderly and oldest adults.

## Data Availability

Data available upon reasonable request.
